# Vitamin B_12_ Prevents Cimetidine-Induced Androgenic Failure and Damage to Sperm Quality in Rats

**DOI:** 10.3389/fendo.2019.00309

**Published:** 2019-07-10

**Authors:** Flávia Luciana Beltrame, Fabiane de Santi, Vanessa Vendramini, Regina Elizabeth Lourenço Cabral, Sandra Maria Miraglia, Paulo Sérgio Cerri, Estela Sasso-Cerri

**Affiliations:** ^1^Department of Morphology and Genetics, Federal University of São Paulo (UNIFESP/EPM), São Paulo, Brazil; ^2^Laboratory of Histology and Embryology, Department of Morphology, Dental School – São Paulo State University (UNESP/FOAr), Araraquara, Brazil

**Keywords:** cimetidine, vitamin B_12_, sperm quality, spermiogenesis, androgenic dysfunction, DNA damage

## Abstract

Cimetidine, used as an anti-ulcer and adjuvant treatment in cancer therapy, causes disorders in the male reproductive tract, including steroidogenesis. However, its effect on sperm quality and male fertility has been poorly addressed. Since vitamin B_12_ has demonstrated to recover spermatogonia number and sperm concentration in cimetidine-treated rats, we evaluated the impact of cimetidine on sperm quality and fertility potential and whether vitamin B_12_ is able to prevent the harmful effect of this drug on steroidogenesis and sperm parameters. Adult male rats were treated for 52 consecutive days as follows: cimetidine group (100 mg/kg of cimetidine), cimetidine/vitamin B_12_ group (100 mg/kg of cimetidine + 3 μg vitamin B_12_), vitamin B_12_ group (3 μg vitamin B_12_) and control group (saline). Serum testosterone levels and immunofluorescence associated to western blot for detection of 17β-HSD6 were performed. Sperm morphology and motility, mitochondrial activity, acrosome integrity, DNA integrity by Comet assay, lipid peroxidation as well as fertility potential were analyzed in all groups. Apoptotic spermatids were also evaluated by caspase-3 immunohistochemistry. In the cimetidine-treated animals, reduced serum testosterone levels, weak 17β-HSD6 levels and impaired spermiogenesis were observed. Low sperm motility and mitochondrial activity were associated with high percentage of sperm tail abnormalities, and the percentage of spermatozoa with damaged acrosome and DNA fragmentation increased. MDA levels were normal in all groups, indicating that the cimetidine-induced changes are associated to androgenic failure. In conclusion, despite the fertility potential of rats was unaffected by the treatment, the sperm quality was significantly impaired. Therefore, considering a possible sperm-mediated transgenerational inheritance, the long term offspring health needs to be investigated. The administration of vitamin B_12_ to male rats prevents the androgenic failure and counteracts the damage inflicted by cimetidine upon sperm quality, indicating that this vitamin may be used as a therapeutic agent to maintain the androgenic status and the sperm quality in patients exposed to androgen disrupters.

## Introduction

Over the years, pharmacological research has made increasing contributions in the area of reproductive medicine. Medications can negatively impact male reproduction, causing changes in sperm quality, infertility, and adverse progeny outcomes. Endocrine disrupters, for example, affect the testes, disrupting Leydig and/or Sertoli cells function and spermatogenesis. These drugs can also harm the epididymal function and sperm maturation (1).

Cimetidine, an anti-ulcer drug widely prescribed over the past 30 years, is an antagonist of histamine H_2_ receptors (2). Nowadays, cimetidine has also been used as an adjuvant therapy in some types of cancer treatments, by exerting immunomodulatory and antiangiogenic effects (3, 4). However, it has been reported in men that this drug causes adverse effects related to antiandrogenic action, such as: loss of libido, impotence, gynecomastia, changes in the serum testosterone levels and decrease in sperm concentration (5, 6). In rodents, cimetidine causes reduction in the serum testosterone levels (7, 8) associated to Leydig cell apoptosis and reduced steroidogenesis (7). This androgenic dysfunction has been associated with germ cell loss and Sertoli cell apoptosis (9–12). The treatment has also induced epididymal androgenic dysfunction due to reduction of SHBG levels and impairment in the epithelial AR nuclear translocation (8). Structural changes caused by cimetidine have also been reported in adult rat vas deferens (13). Some studies have demonstrated the impact of cimetidine on sperm count and motility (12, 14, 15), but the effect of this drug on other sperm parameters and male fertility has not yet been addressed.

Vitamin B_12_, or cobalamin, is an essential nutrient found in foods of animal origin, including dairy products, and has been useful for the treatment of peripheral nerve injury (16) and neuropathic pain (17). This vitamin also plays an important role in DNA synthesis and cell division (18), being an essential nutrient for the maintenance of testicular function and normal fertility [revised by Banihani (19)]; the therapeutic effects of this vitamin have been demonstrated in rats with damaged spermatogenesis and sperm parameters (20, 21). Vitamin B_12_ is able to recover the seminiferous epithelium of animals treated with cimetidine (11), increasing the number of spermatogonia and spermatocytes, and ameliorating the sperm concentration (12). A potential effect of vitamin B_12_ on the spermatogenic recovery has also been demonstrated in animals receiving co-administration of the antineoplastic busulphan and vitamin B_12_ (22, 23).

For years, traditional methods for evaluation of semen quality, such as sperm count and morphology, have been usually used to assess male fertility potential and conception (24). However, additional tests have been developed to provide more information on the fertilizing potential of spermatozoa since DNA damage and changes in packaging of sperm chromatin may affect the expression of paternal genes and the embryonic development (25).

Cimetidine has exerted an androgenic disrupter effect in testes (7, 9–12), vas deferens (13) and epididymis (8). However, the effect of this drug on the sperm quality and fertility potential needs to be clarified. In this study, we evaluated the impact of cimetidine on the sperm quality parameters and fertility potential. Since vitamin B_12_ has prevented the harmful effect of cimetidine on spermatogenesis, we also evaluated whether this vitamin is able to prevent the antiandrogenic effect of cimetidine on steroidogenesis and sperm parameters.

## Materials and Methods

### Animals and Experimental Groups

One-hundred-day-old male (*n* = 56) and ninety-day-old female (*n* = 55) Holtzman rats were maintained in polypropylene cages under 12-h light/12-h dark cycle at a controlled temperature (23 ± 2°C), with water and food provided *ad libitum*.

The male rats were distributed into four groups: cimetidine (CMTG; *n* = 14), cimetidine/vitamin B_12_ (CMT/B_12_G; *n* = 14), vitamin B_12_ (B_12_G; *n* = 14) and control group (CG; *n* = 14). The animals from CMTG received daily intraperitoneal injections (ip) of 100 mg of cimetidine (Hycimet^®^, 300 mg—Hypofarma, MG, Brazil) per kg. This dosage does not cause systemic toxicity and was based on the therapeutic dose range of cimetidine, usually administered to humans. Allometric extrapolation of the dosage was performed to rats by the exponent 0.75 (BW^3/4^ scaling), taking into account body mass and basal metabolic rate (26).

The animals from CMT/B_12_G received ip injections containing a solution of 100 mg/kg of cimetidine and 3 μg of vitamin B_12_-cianocobalamin (Citoneurin^®^, 5,000 μg—Merck S.A., Brazil). This dosage of vitamin was determined from a preliminary evaluation of the amount of daily vitamin B_12_ intake with the food by the animals at this age (11). The animals from B_12_G received only vitamin (3 μg/day) and the control animals received saline solution, corresponding to the volume of CMTG. All animals received the treatment for 52 consecutive days, a period that corresponds to a complete spermatogenic cycle in rats (27), and that induce testicular changes (7, 11, 28). According to studies using rodents treated with other drugs, this period corresponds to a long term treatment in rats (29). It is important to emphasize that, clinically, patients with gastric and/or colon cancer may be treated with cimetidine for up to 2 years (30).

After the treatment, the animals were anesthetized with 80 mg/Kg of ketamine hydrochloride (Francotar; Virbac Brazil Ind. Com. Ltda) and 8 mg/Kg of xylazine hydrochloride (Virbaxyl; Virbac Brazil Ind. Com. Ltda). Blood samples were collected via cardiac puncture with BD Vacutainer^®^ Blood Collection Tubes (SSTII Plus, BD Biosciences) for serum testosterone measurement. Testicular fragments were collected for histological analysis or frozen at −80°C for western blot and lipid peroxidation analysis. Sperm samples were collected from cauda epididymis for the analysis of motility, morphology, mitochondrial activity, acrosome and DNA integrity, and TBARS (Thiobarbituric Acid Reactive Substances) test. After sperm collection, the animals were euthanized by overdose of anesthesia. The other 24 males and the female rats were used for fertility test.

### Serum Testosterone Measurement

Serum testosterone levels were determined by chemiluminescence immunoassay by using Access^®^ 2 Immunoassay System (Beckman Coulter, CA, USA) and the Access Testosterone Immunoassay kit (Beckman Coulter, CA, USA). The antibody was specific for testosterone (≤2% cross-reactivity) and the analytical sensitivity was 10 ng/dL. The analyses were performed at São Lucas Clinical and Microbiological Laboratory (Araraquara, SP, Brazil).

### Histological Procedures

The testes were removed and fixed for 48 h at room temperature in 4% freshly prepared paraformaldehyde (MERK, Germany) buffered with 0.1M sodium phosphate (pH 7.4). Subsequently, the testes were dehydrated in graded ethanol and embedded in glycol methacrylate (Historesin Embedding Kit, Jung, Germany) or paraffin. The historesin sections (3 μm) were stained with Gill's Hematoxylin and Eosin (H.E) for morphological analyses. The paraffin sections (6 μm thick) were adhered to silanized slides and subjected to the immunohistochemistry reaction for detection of activated caspase-3 and immunofluorescence reaction for detection of 17β-hydroxysteroid dehydrogenase 6 (17β-HSD6), enzyme involved in steroidogenesis.

### Immunohistochemistry and Immunofluorescence Reactions

Paraffin testicular sections adhered to silanized slides were immersed in 0.001 M sodium citrate buffer (pH 6.0) and maintained at 90°C in a microwave oven for antigen recovery. For the immunohistochemistry reaction, the slides were submitted to inactivation of endogenous peroxidase with 9% hydrogen peroxide. The sections were incubated in 2% BSA to block non-specific binding and washed in phosphate-buffered saline (PBS; pH 7.4). The slides were incubated overnight in a humidified chamber at 4°C with the following primary antibodies: rabbit anti-activated caspase-3 antibody (Abcam; Cambridge, UK; ab-4051), diluted 1:100 (7); and rabbit anti-17β-HSD6 antibody (Santa Cruz Biotechnology Inc., Dallas, USA; sc-101878), at a concentration of 2 μg/ml, according to Beltrame et al. (7) and Sasso-Cerri et al. (23). The sections incubated with anti-activated caspase-3 antibody were washed in PBS and incubated at room temperature with Labeled StreptAvidin-Biotin Kit (Universal Dako LSAB, Dako Inc., Carpinteria, CA, USA), and the reaction was revealed with 3,3′-diaminobenzidine (DAB, Sigma-Aldrich, USA). After washing in tap water, the sections were counterstained with Carazzi's hematoxylin. The images were captured using a DP-71 Olympus camera attached to an Olympus BX-51 light microscope. The sections incubated with anti-17β-HSD6 antibody were washed in PBS and incubated with Alexa Fluor^®^ 594 goat anti-rabbit IgG antibody ReadyProbes^®^ reagent (Life Technologies, Calrsbad, USA) for 30 min at room temperature. The nuclear staining was performed with DAPI (Molecular Probes by Life Technologies; Carlsbad, California, USA). The analyses were performed using a fluorescent microscope DM400 B LED, a camera DFC-550 and an Image Analysis System LAS4 (Leica Microsystems, Wetzlar, Germany).

Sections used as negative controls were incubated with non-immune serum in place of primary antibodies.

### 17β-HSD6 Immunofluorescent Area

The analysis of the immunofluorescence was performed using a DFC 550 Camera (Leica) attached to a BM4000 B LED microscope (Leica), and the Leica Application Suite software (LAS 4.3, Leica). The measurement of 17β-HSD6 immunoexpression was performed in two non-serial testicular sections per animal from the CG, B_12_G, CMTG and CMT/B_12_G (*n* = 5/group). In the sections, the 17β-HSD6 immunofluorescence was measured in a standardized interstitial tissue area of 120,000 μm^2^ per animal at x220, and the immunofluorescent area per μm^2^ of interstitial tissue was obtained. All the parameters of the software, including threshold adjustment and color range—hue, saturation and intensity (LAS 4.3, Leica) were calibrated and standardized for each image analyzed.

### Western Blot Assay

Since 17β-HSD is a specific enzyme present only in Leydig cells (LC), fragments of the whole testes (containing interstitial tissue/LC and seminiferous tubules) was assessed for detection of the levels of this enzyme by western blot. Protein extraction was performed using lysis buffer [50 mM Tris pH 8.0, 150 mM NaCl, 1 mM EDTA, 10% glycerol, 1%Triton X-100, 1 mM phenylmethylsulfonyl fluoride (PMSF)], containing 5 ng/mL of the following protease inhibitors: Pepstatin, Leupeptin, Aprotinin, Antipain, and Chymostatin (Sigma-Aldrich, St. Louis, USA; P8340). After tissue homogenization using Polytron PT 1600E (Kinematica, Luzernerstrasse, Switzerland), the samples were centrifuged at 8,944 × g (20 min, 4°C), and the supernatant was collected. Bradford assay (Sigma-Aldrich, St. Louis, USA; B6916) was performed to determine protein concentration. Protein samples were separated in 10% SDS-PAGE and transferred to a nitrocellulose membrane (GE Healthcare, Little Chalfont, UK). The membranes were treated with 5% non-fat dry milk diluted in PBS/T (PBS/0.05% Tween 20) for 1 h for nonspecific blocking and incubated overnight at 4°C with rabbit anti-17β-HSD6 polyclonal antibody (0.5 μg/mL; Santa Cruz Biotechnology Inc., Dallas, USA; sc-101878). The membranes were washed in PBS/T and incubated for 1 h at room temperature with HRP conjugated anti-rabbit secondary antibody (2.2 μg/mL; Sigma-Aldrich, St. Louis, USA). The reactions were detected using enhanced chemiluminescence system (ECL). The membranes were incubated with stripping buffer (1,5% glycine, 0,1% SDS, 1% Tween-20) for 10 min at room temperature, washed in PBS and reprobed with rabbit anti-actin antibody (0.09 μg/mL; Sigma-Aldrich, USA) for positive control. For all the groups, the assays were reproduced in triplicate. The optical density of protein bands was analyzed using ImageJ (version 1.50i) according to the NIH instructions for Gel analysis. For background subtraction, the baseline of each peak was defined and the area under the curve was measured. The protein levels were normalized to actin.

### Sperm Morphology and Motility

The epididymis was dissected, and a small cut was made in the cauda epididymis with a razor blade. An aliquot (3 μl) of sperm was placed in 4 mL of distilled water to immobilize spermatozoa. A sample drop was smeared onto a slide and stained by Shorr method. The images of two hundred spermatozoa were captured by a DP-71 Olympus camera attached to a light microscopy (BX-51, Olympus, Japan) and a software Image-Pro Express 6.0 (Olympus). Spermatozoa showing sperm head and tail alterations were quantified, at x1380, according to Filler (31) and Miranda-Spooner et al. (32).

For the motility analysis, the same cauda epididymis was placed into a petri dish containing Hank's Balanced Salt Solution (HBSS) medium, supplemented with 0.2% bovine serum albumin (BSA), and incubated at 37°C. Subsequently, 50 μL were smeared onto a slide and evaluated under light microscopy at × 1,000. Two-hundred spermatozoa were evaluated and the ratio of motile sperm to the total number of sperm was obtained. The motile sperm were classified according to the type of movement, based on the World Health Organization category (33, 34): fast progressive, slow progressive and nonprogressive.

### Mitochondrial Activity

The sperm samples were collected and prepared as described previously (35). The mitochondrial activity was evaluated according to the method proposed by Hrudka (36) and as described by Mendes et al. (37), with minor modifications. A 50 μL semen aliquot was incubated with 200 μL of a medium containing 3–3′ Diaminobenzidine (DAB, Sigma-Aldrich^®^, MO, USA) at 1 mg/mL of phosphate-buffered saline (PBS) (0.15 M, pH 7.2) at 37°C, in a dark room, for 1 h. Following incubation, the samples were smeared onto a slide and fixed in 10% formaldehyde (prepared from paraformaldehyde). The images were captured using a DP-71 Olympus camera attached to a light microscope (Olympus, BX-51, Tokyo, Japan) and a software Image-Pro Express 6.0 (Olympus). Two-hundred spermatozoa were analyzed at × 1,380. The spermatozoa were evaluated and classified according to the mid-piece stain, and the rate of cytochemical activity (RCA) was obtained.

### Acrosome Integrity

The acrosome integrity was assessed by a staining method with Peanut Agglutinin (PNA), according to Varisli et al. (38), with few modifications. To determine the acrosome integrity, PNA Lectin from *Arachis hypogaea* (peanut) conjugated with Alexa Fluor^®^ 488 (Molecular Probes^®^, Inc., OR, USA) was used. This lectin is specific for terminal β-galactose, present in the acrosome. Sperm samples were collected and stored as described previously by Vendramini et al. (35). For the analysis, the samples were thawed and 100 μL were incubated in dark tubes containing 150 μL of PNA-Alexa Fluor^®^ 488 (20 μg/mL), at 37°C for 30 min. After the incubation, 100 μL of mixture was smeared on glass slide for microscopy and analyzed under a fluorescent microscope DM400 B LED (Leica, Germany). Two hundred spermatozoa were analyzed at × 545 and classified as intact acrosome (spermatozoa exhibiting intense and moderate bright fluorescence in the acrosome region) or damaged acrosome (spermatozoa presenting weak, irregular, or absent fluorescence in the acrosome region).

### Lipid Peroxidation in Testicular Homogenate and Epididymal Sperm (Thiobarbituric Acid Reactive Substances Test—TBARS)

The levels of lipid peroxidation in the testis and epididymal sperm were assessed by the dosage of malondialdehyde (MDA) used as an oxidative stress marker. The TBARS assay is based on the reaction of MDA with thiobarbituric acid (TBA) to form a 1:2 adduct (39).

The MDA content in testicular homogenate was determined by a chemical colorimetric method using a MDA-TBA assay kit [Lipid Peroxidation (MDA) Assay Kit; Sigma-Aldrich^®^, MO, USA]. The assay was performed in accordance with the manufacturer's instructions and the absorbance was measured at 532 nm (Synergy H1 Hybrid Multi-Mode Microplate Reader; BioTek Instruments, Inc., VT, USA). The concentrations of MDA in each sample was estimated from a standard curve and expressed as malondialdehyde nmol/g tissue.

For the detection of lipid peroxidation in epididymal sperm, the protocol was modified from Aitken et al. (40) and Gomez et al. (41). In order to make sensitive measurements of MDA on spermatozoa, the stimulation of a lipid peroxidation cascade with a ferrous ion promoter was necessary. For this assay, 250 μL sperm suspension at a concentration of 40 × 10^6^ cells/mL were incubated with 125 μL ferrous sulfate (4 mM) and 125 μL ascorbic acid (20 mM) at 37°C for 2 h. After cooling the samples in an ice bath, 250 μL of this reaction mixture was supplemented with 500 μL 10% chilled trichloroacetic acid (TCA) and centrifuged at 18,000 × g (15 min, 15°C). Two-hundred μL of the supernatant was added to a mixture containing 8.1% sodium dodecyl sulfate (SDS), 20% acetic acid solution (pH 3.5), 0.8% aqueous solution of TBA (cleared with 0.05N NaOH) and distilled water (42). The reaction mixture was incubated at 95°C for 1 h, cooled in ice bath and pipetted into a 96 well black-plate for analysis. The fluorescence intensity was measured on a fluorescence multiwell plate reader (Synergy H1 Hybrid Multi-Mode Microplate Reader; BioTek Instruments, Inc., VT, USA) with excitation wavelength at 515 nm and emission at 553 nm. The results were estimated from a standard curve generated by incubating serial dilutions of a MDA Standard Solution (Sigma-Aldrich^®^, MO, USA) and expressed as malondialdehyde nmol/10^7^ spermatozoa.

### Comet Assay

Sperm DNA damage were analyzed by the alkaline Comet assay, as described by Vendramini et al. (35), with minor modifications.

Samples containing spermatozoa stored at −80°C were thawed at 37°C for 2 min in a water bath and diluted in prewarmed 0.5% low melting point (LMP) agarose (Sigma-Aldrich^®^, MO, USA). This solution was placed onto slides precoated with 1% normal melting point agarose (Agargen^®^ Madrid, Spain) and the slides were stored at 4°C for 15 min. To avoid further damage to the sperm DNA, the next steps were performed in the dark. The slides were covered with chilled lysis buffer, containing dithiothreitol at 40 mmol/L (Sigma-Aldrich^®^, MO, USA), and incubated for 60 min at 4°C. Subsequently, a second lysis buffer, prewarmed (37°C) and containing proteinase K at 100 mg/mL (Sigma-Aldrich^®^, MO, USA), was used to cover the slides for 2.5 h in a 37°C incubator. The slides were then gently washed in chilled distilled H_2_O, covered with freshly prepared alkaline solution (pH 12.1) for 45 min and placed into a horizontal electrophoresis box (Bio-Rad Laboratórios Brasil Ltda; São Paulo, Brazil) filled with Tris/borate/EDTA (TBE) buffer, and submitted to electrophoresis at 30 V for 10 min.

For the analysis, the slides were stained with ethidium bromide and observed under a fluorescent microscope DM400 B LED (Leica, Germany) containing a camera DFC-550 (Leica, Germany). One hundred comets were analyzed per animal by using LUCIA Comet Assay Analysis^®^ v. 7.30 software (Prague, CZ). The parameters evaluated included: tail DNA percentage, tail length, tail moment (tail length × tail DNA %/100) and Olive moment (tail DNA % × the distance between the centers of mass of head and tail regions).

### Fertility Evaluation

Twenty-four male rats (*n* = 6/group) were mated overnight with two nulliparous females in natural proestrous/estrous. The following morning, the presence of spermatozoa was checked in the vaginal smears, and the day of sperm detection in the vaginal smear was considered gestational day 1 (GD 1). On the 20th day of gestation (GD 20), the females were euthanized, and uterus and ovaries were collected. The number of corpora lutea, implants, resorptions and live fetuses was recorded, and the following parameters were calculated: (1) fertility potential (number of implantation sites/number of corpora lutea × 100); (2) rate of pre-implantation loss (number of corpora lutea–number of implantations/number of corpora lutea × 100); and (3) rate of post-implantation loss (number of implantations–number of live fetuses/number of implantation × 100) (35, 43). The weights of live fetuses and placenta were also obtained.

### Statistical Analysis

Statistical analysis of data was performed using the GraphPad Prism^®^ 6.0 software (GraphPad Software, CA, USA). All data were checked for normal distribution using the D'Agostino-Pearson normality test. The homogeneity of variance was tested using the Bartlett's or Brown-Forsythe tests. Normally distributed data were presented as mean ± SD. Non-normally distributed data were logarithmically transformed. In case of persistent non-normality, non-parametric test was used and the data were presented as median and interquartile intervals.

The parametric variables submitted to the one-way ANOVA (testosterone measurement, 17β-HSD6 immunofluorescent area, optical density of protein bands, percentage of motile spermatozoa, rate of cytochemical activity, acrosome integrity, levels of lipid peroxidation in the testis and epididymal sperm, parameters of Comet assay, and the following fertility parameters: corpora lutea, implantations and live fetuses number, and fetus and placental weights) and to the two-way ANOVA (sperm morphology, quality of sperm movement and classification of mitochondrial activity) were followed by Tukey's *post-hoc* test.

Non-parametric data (fertility potential, pre- and post-implantation losses and resorptions number) were compared by Kruskal-Wallis test (followed by Dunn's *post-hoc* test when necessary). The significance level considered for all tests was *p* < 0.05.

## Results

### Testicular Histology and 17β-HSD6 Immunoexpression

**Figure 1:**
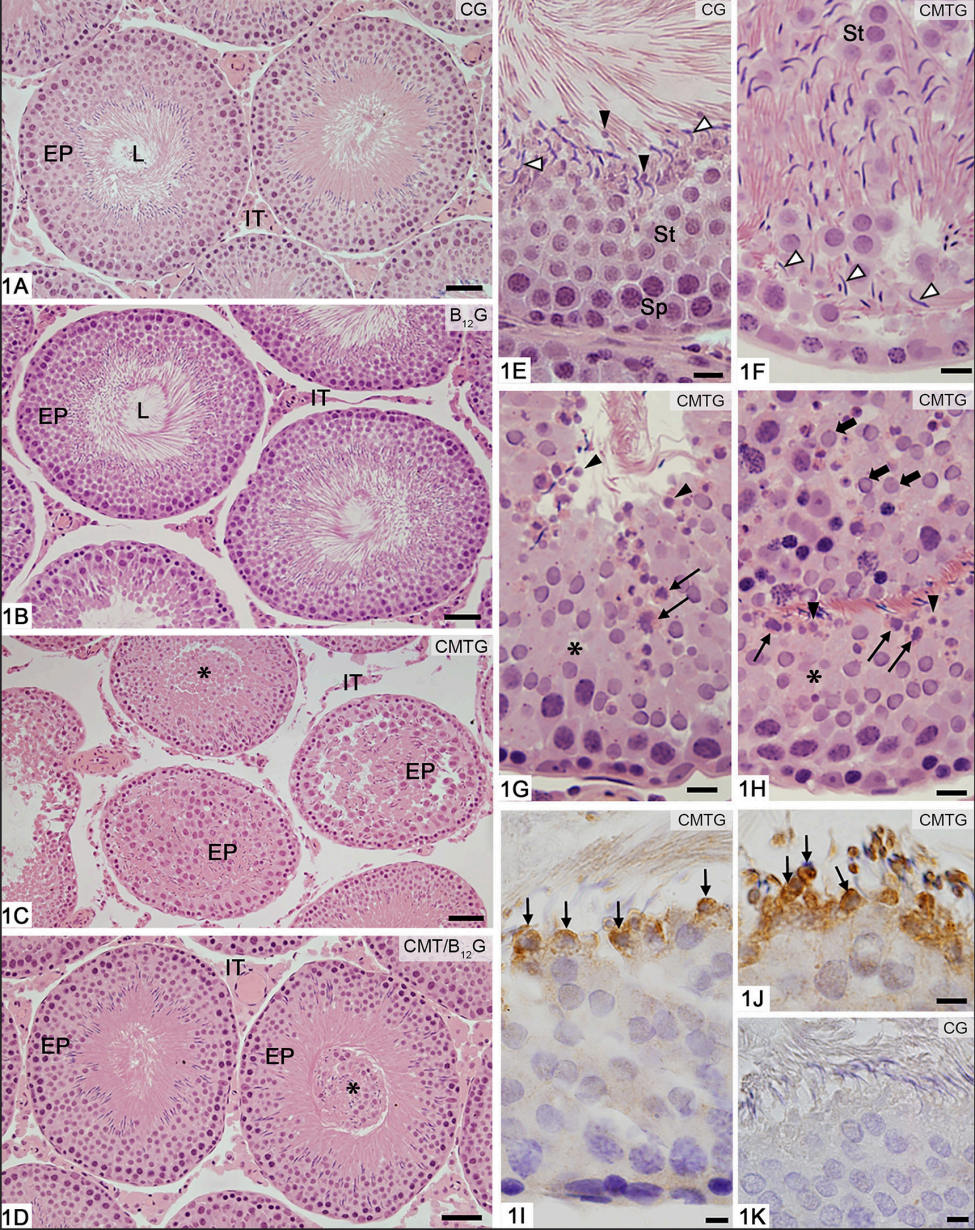
Photomicrographs of seminiferous tubules stained by H.E **(A–H)** and submitted to immunohistochemistry reaction for detection of activated-caspase-3 **(I–K)**. In **(A,B)**, the seminiferous tubule sections show normal epithelial integrity (EP) and absence of germ cells in the tubular lumen (L). In **(C)**, damaged seminiferous tubules show epithelial disorganization (EP) and detached germ cells filling the tubular lumen (asterisk). In **(D)**, except for the presence of sloughed germ cells filling the tubular lumen (asterisk), the seminiferous tubules (EP) show normal integrity similarly to CG and B_12_. IT, interstitial tissue. In **(E–H)**, portions of seminiferous epithelium at androgen-dependent stages of rats from CG and CMTG under high magnification. In **(E)**, note the normal integrity of the seminiferous epithelium, in which spermatocytes (Sp), round (St) and elongate (white arrowheads) spermatids are organized in concentric layers. Residual bodies are also observed (black arrowheads). In **(F)**, the disorganized epithelium shows round (St) and elongated (arrowheads) spermatids abnormally distributed. In **(G,H)**, the epithelium shows lack of spermatids (asterisks) and abnormal spermatids with condensed chromatin, suggesting apoptosis (thin arrows). Residual bodies are observed (arrowheads). In **(H)**, numerous sloughed round spermatids are seen in the tubular lumen (thick arrows). In **(I,J)**, portions of tubules at androgen-dependent stages show caspase-3 immunolabeled (brown-yellow color) spermatids (thin arrows), confirming apoptosis. In K (control group), immunolabeled germ cells are not found. Bars: 37 μm **(A–D)**, 18 μm **(E–H)**, 7 μm **(I–K)**.

In the animals from CG and B_12_G, the seminiferous tubules showed normal aspect, with germ cells organized in concentric layers and absence of germ cells in the tubular lumen (Figures 1A,B,E). On the other hand, seminiferous tubules at androgen-dependent stages (VII-VIII) showing epithelial disorganization and detached germ cells (mainly spermatids) in the tubular lumen were found in CMTG (Figures 1C,H). Depletion of round spermatids, round spermatids with abnormal features and elongated spermatids abnormally distributed in the epithelium were observed (Figures 1F–H). Caspase-3-positive spermatids were also found in CMTG (Figures 1I,J), but not in CG (1K). Although tubules with sloughed germ cells in the lumen were also found in CMT/B_12_G, the seminiferous epithelium histoarchitecture was preserved in the animals from this group (Figure 1D).

**Figure 2:**
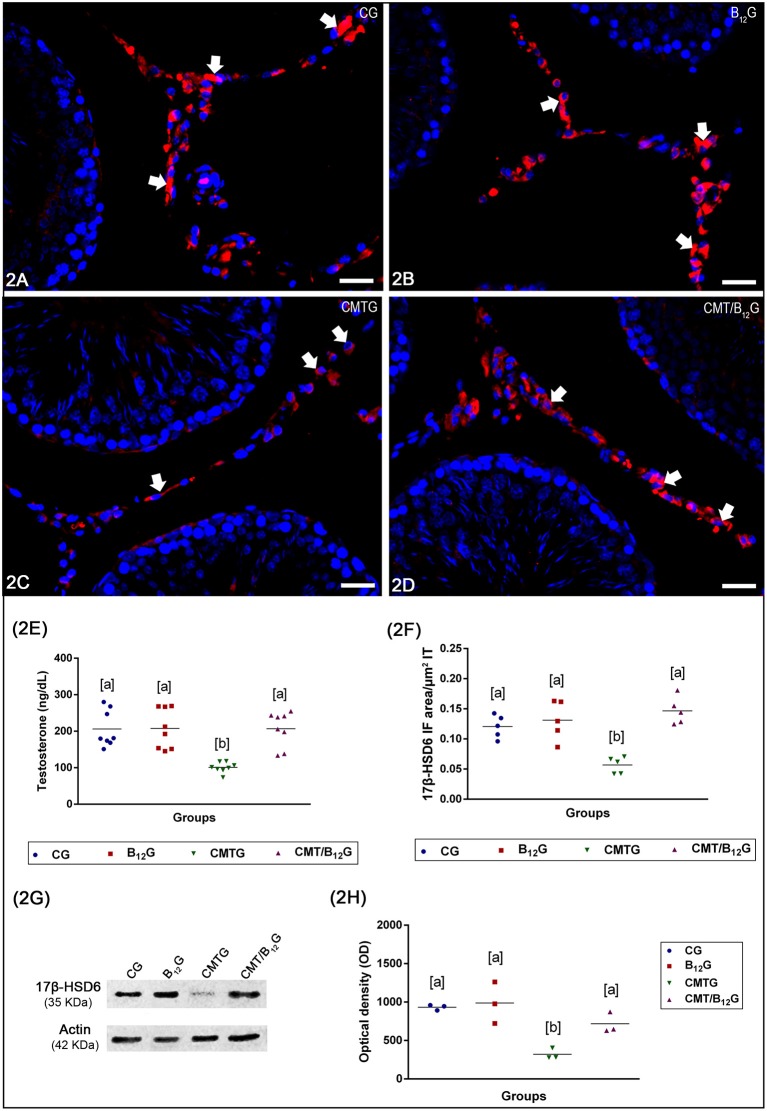
**(A–D)** Photomicrographs of testicular sections submitted to immunofluorescence for detection of 17β-HSD6. In CG, B_12_G and CMT/B_12_G **(A,B,D)**, the Leydig cells show strong immunofluorescence (arrows) in comparison with CMTG **(C)**. Bars: 36 μm. In **(E)**, the testosterone level in CMTG is significantly reduced when compared to the other groups (Values expressed as mean ± SD. One-way ANOVA followed by Tukey's test; a ≠ b; *p* < 0.05; *n* = 8). In **(F)**, the 17β-HSD6 immunofluorescent area per μm^2^ of interstitial tissue of animals from CMTG is significantly reduced when compared to the other groups (Values expressed as mean ± SD. One-way ANOVA followed by Tukey's test; a ≠ b; *p* < 0.05; *n* = 5). In **(G,H)**, Western blot analysis of 17β-HSD6 levels in testicular extracts. Strong bands at 35 kDa, corresponding to the 17β-HSD6 molecular weight, are detected in the CG, B_12_G and CMT/B_12_G while a weak band is detected in CMTG. Actin signal is observed in all groups. 17β-HSD6 optical density (OD) shows high 17β-HSD6 levels in the CG, B_12_G and CMT/B_12_G and low 17β-HSD6 levels in the CMTG (Values expressed as mean ± SD. One-way ANOVA followed by Tukey's test; a ≠ b; *p* < 0.05; *n* = 3).

The immunoexpression of 17β-HSD6 in the Leydig cells of the animals from CMTG reduced significantly when compared to CG, B_12_G and CMT/B_12_G (Figures 2A–D, F). However, in the cimetidine-treated animals supplemented with vitamin B_12_ (CMT/B_12_G), the immunofluorescence was similar to CG and B_12_G (Figures 2D,F). The analysis by western blot (tudo minúsculo), including the optical density analysis, confirms the results obtained by immunofluorescence (Figures 2G,H).

### Serum Testosterone Levels

As shown in Figure 2E, serum testosterone levels decreased significantly in CMTG when compared with CG (51%) and B_12_G (55%). A significant increase (2 fold) was observed in the CMT/B_12_G when compared with CMTG. No statistical difference was detected among the animals from CG, B_12_G and CMT/B_12_G groups.

### Sperm Morphology and Motility

**Figure 3:**
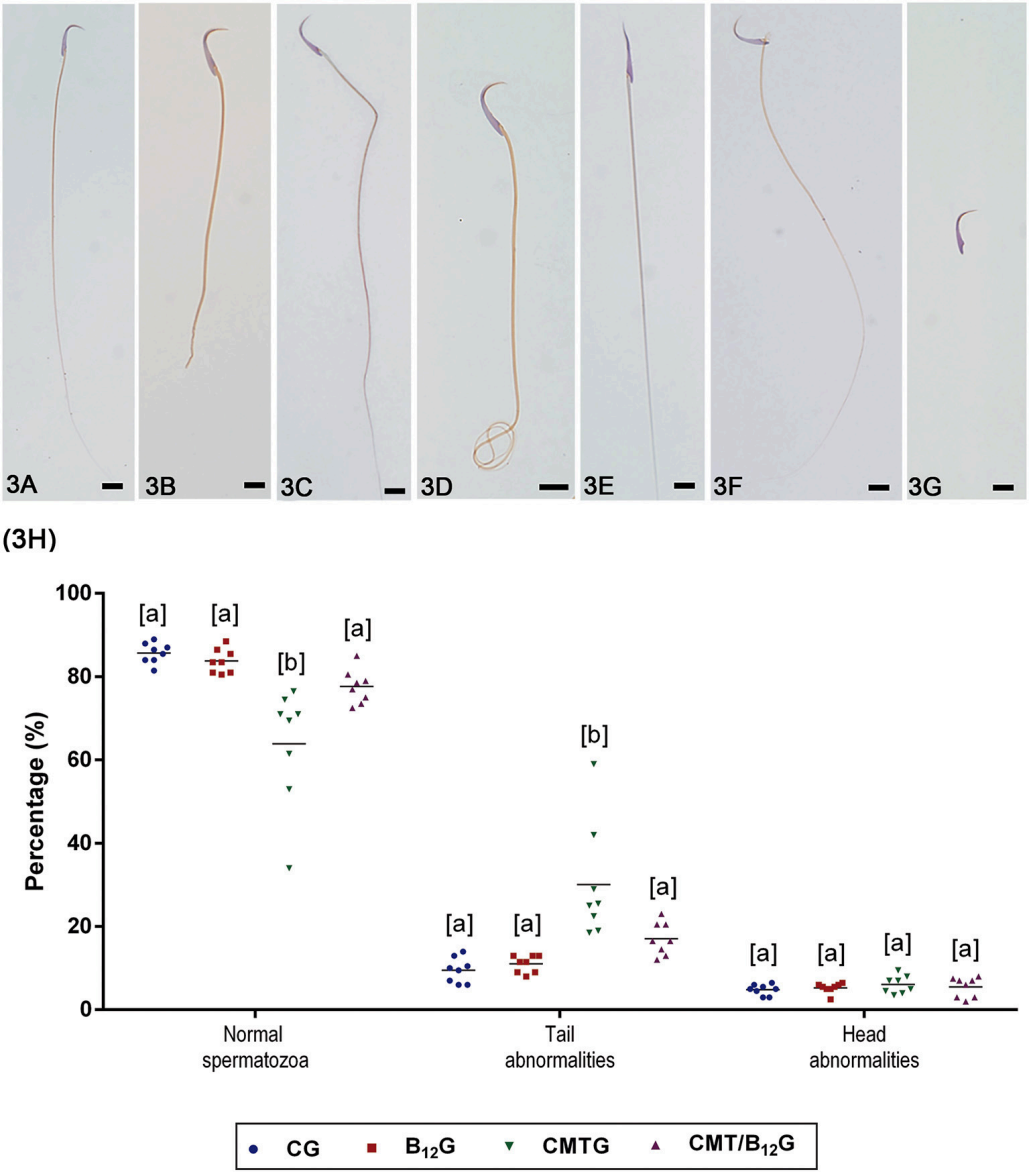
**(A–G)** Representative photomicrographs of spermatozoa classified according to the morphology. In **(A)**, a normal spermatozoon. In **(B–D)**, spermatozoa with abnormal tail (broken, bent, or coiled). In **(E–G)**, spermatozoa with abnormal head (without characteristic curvature or isolated). Bars: 7 μm. In **(H)**, percentage of normal spermatozoa and sperm head and tail morphological abnormalities. (Values expressed as mean ± SD. Two-way ANOVA followed by Tukey's test; a ≠ b; *p* < 0.05; *n* = 8).

The percentage of normal spermatozoa (Figure 3A) decreased significantly in CMTG (Figure 3H). In this group, the percentage of spermatozoa with abnormal tail (broken, bent, or coiled—Figures 3B–D) increased significantly when compared with CG and B_12_G animals. On the other hand, the CMT/B_12_G showed a significant reduction of abnormal spermatozoa (with tail alterations) in comparison with the CMTG (Figure 3H). The percentage of spermatozoa with head morphological alterations (without typical curvature or isolated—Figures 3E–G) was similar among the groups (Figure 3H).

**Figure 4:**
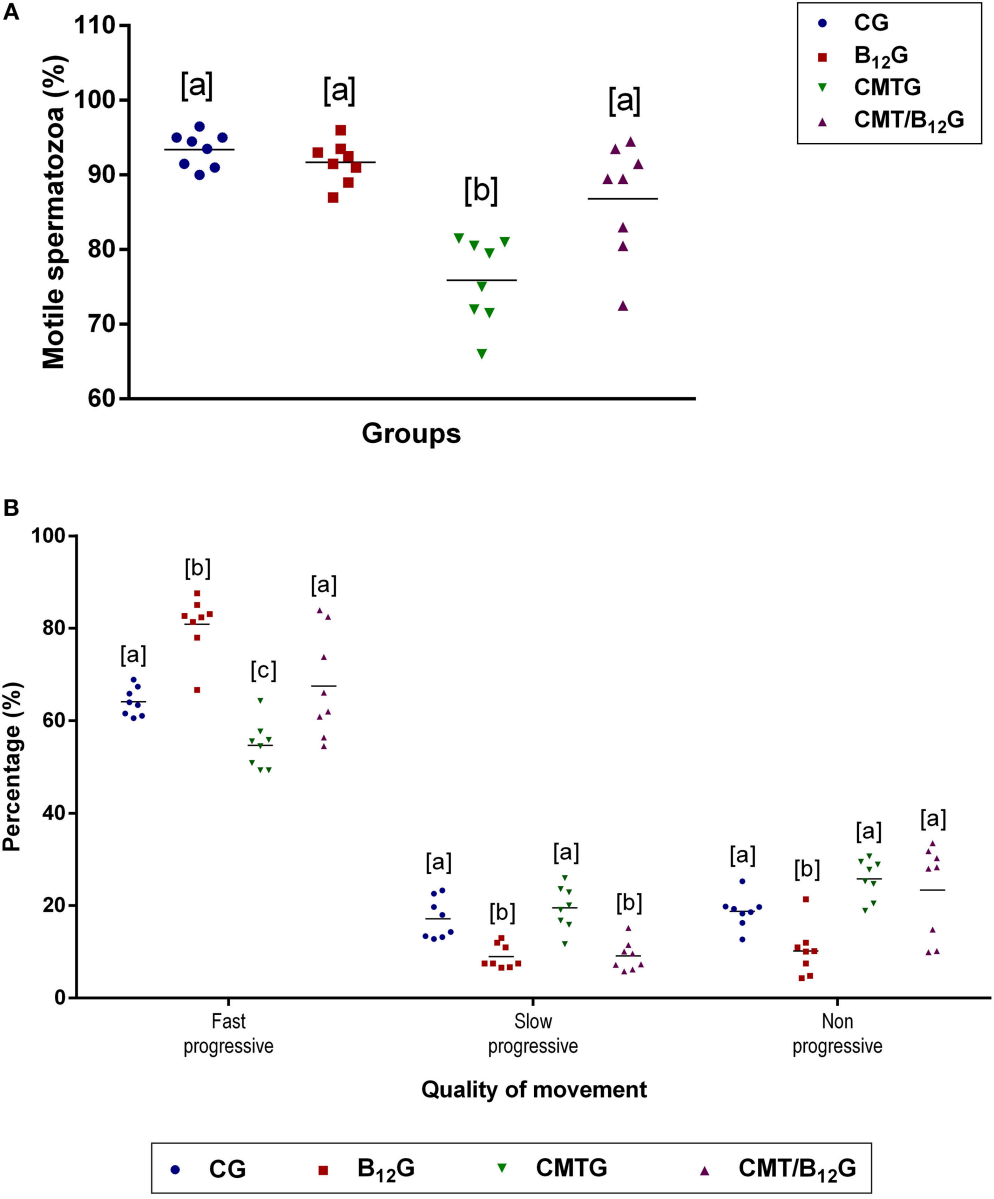
Percentage of motile spermatozoa **(A)** and quality of sperm movement **(B)** in animals from CG, B_12_G, CMTG and CMT/B_12_G. Values expressed as mean ± SD. **(A)** One-way ANOVA followed by Tukey's test (a ≠ b; *p* < 0.05; *n* = 8). **(B)** Two-way ANOVA followed by Tukey's test (a ≠ b ≠ c; *p* < 0.05; *n* = 8).

The percentage of motile spermatozoa in CMTG decreased significantly in comparison with the other groups (Figure 4A). According to the quality of movement, a significant reduction in the percentage of spermatozoa with fast progressive movement was observed in CMTG, when compared with the other groups (Figure 4B). On the other hand, the animals from CMT/B_12_G showed a significant increase in the percentage of spermatozoa with fast progressive movement in comparison with CMTG, and decrease in the percentage of spermatozoa with slow progressive movement (Figure 4B). It is important to emphasize that in the animals that received vitamin only (B_12_G), the percentage of fast progressive spermatozoa was higher whereas the percentage of non-progressive spermatozoa was lower in comparison with the other groups (Figure 4B).

### Mitochondrial Activity

**Figure 5:**
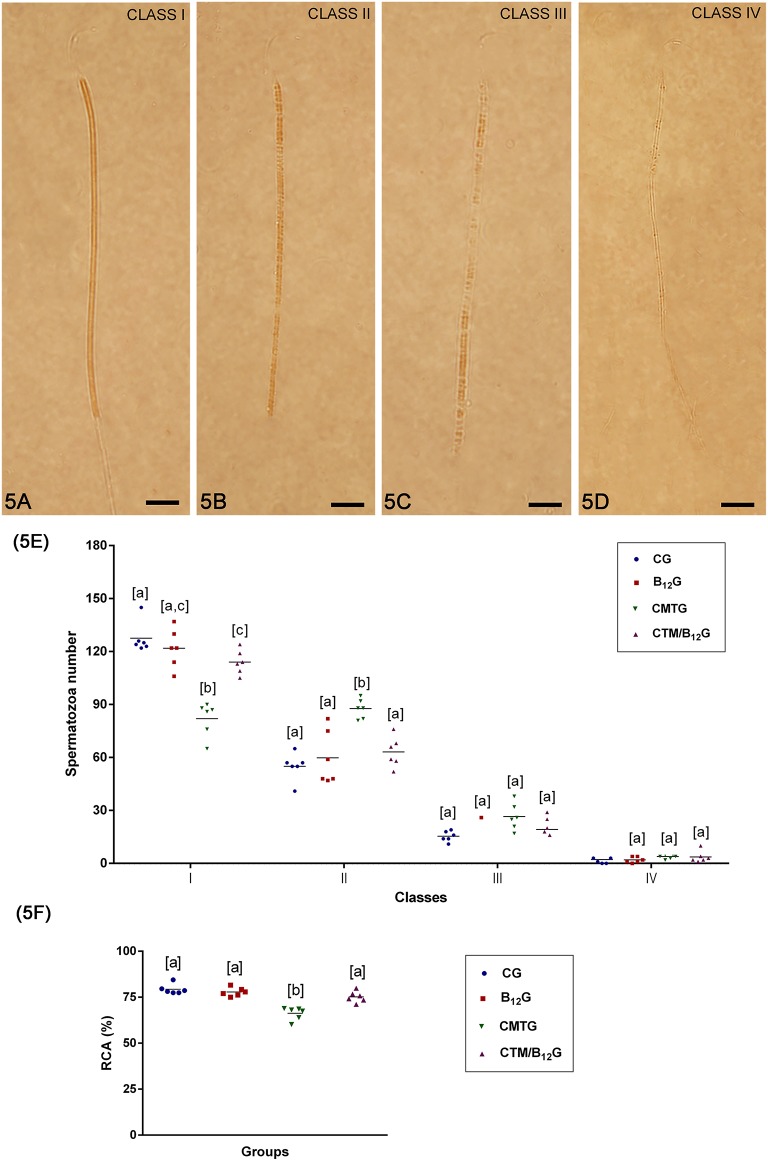
**(A–D)** Classification of the mitochondrial activity. **(A)** Class I spermatozoon, when the mid-piece was totally stained; **(B)** class II spermatozoon, when most of the mid-piece was stained; **(C)** class III spermatozoon, when a part of mid-piece was stained; and **(D)** class IV spermatozoon, when the mid-piece was not stained. Bars: 7 μm In **(E)**, number of spermatozoa per classes of mitochondrial activity (Values expressed as mean ± SD. Two-way ANOVA followed by Tukey's test; a ≠ b ≠ c; *p* < 0.05; *n* = 6). In **(F)**, rate of cytochemical activity (RCA) (Values expressed as mean ± SD. One-way ANOVA followed by Tukey's test; a ≠ b; *p* < 0.05; *n* = 6).

In CMTG, while the percentage of Class I spermatozoa (Figure 5A) was low, Class II spermatozoa (Figure 5B) was higher than the other groups (Figure 5E), resulting in a low rate of cytochemical activity (RCA) (Figure 5F). Otherwise, CMT/B_12_G showed a significant improvement of this qualitative parameter in comparison with CMTG (Figures 5E,F). There was no statistical difference among the groups in the percentage of Classes III and IV spermatozoa (Figures 5C–E).

### Acrosome Integrity

**Figure 6:**
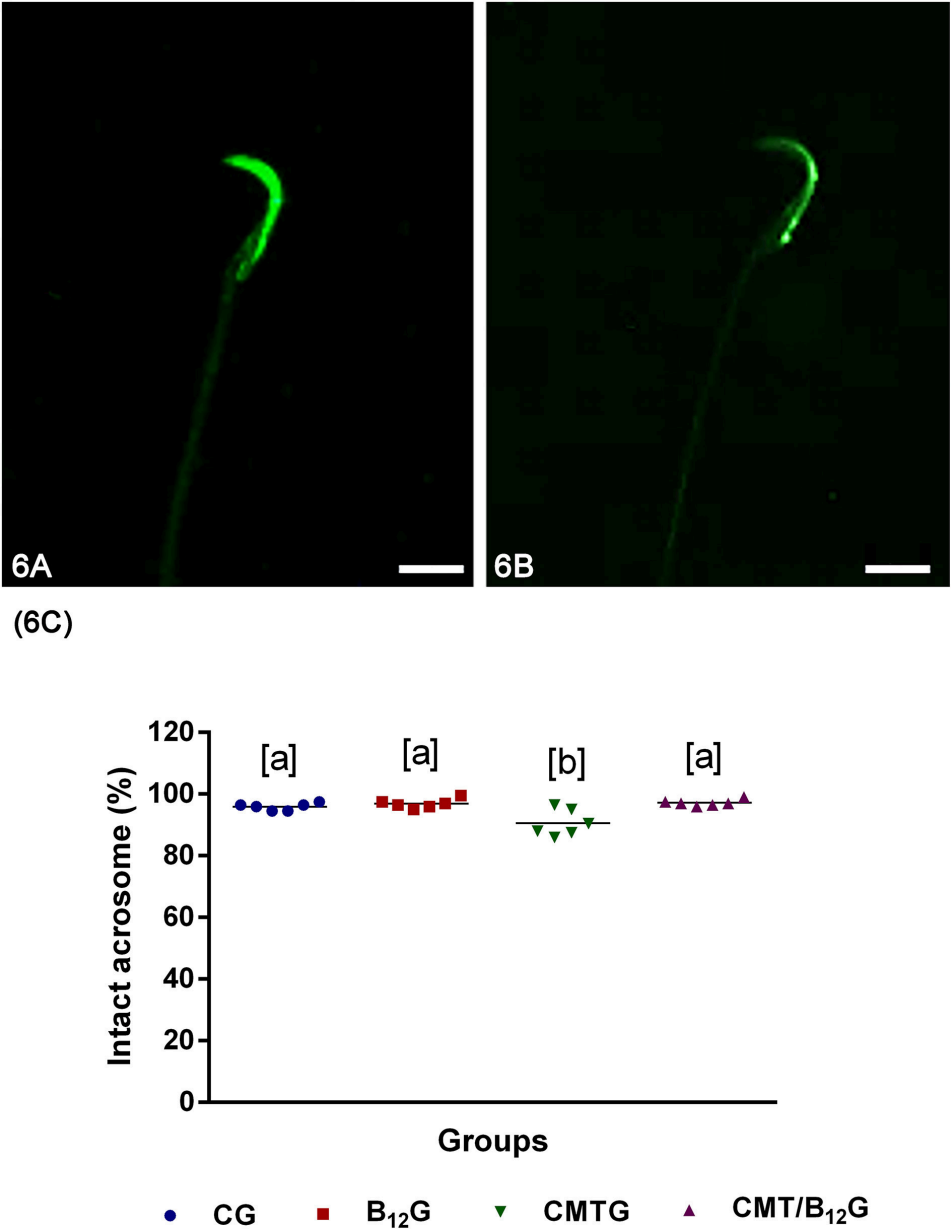
**(A,B)** Representative photomicrographs of spermatozoa classified according to the acrosome integrity. In **(A)**, spermatozoa with intact acrosome; in **(B)**, spermatozoa with damaged acrosome. Bars: 7 μm. **(C)** Percentage of spermatozoa with intact acrosome in the animals from CG, B_12_G, CMTG and CMT/B_12_G. (Values expressed as mean ± SD. One-way ANOVA followed by Tukey's test; a ≠ b; *p* < 0.05; *n* = 6).

According to the analysis of acrosome integrity (intact or damaged), the animals from the CMTG showed a higher number of spermatozoa with damaged acrosome in comparison with the other groups. However, in CMT/B_12_G, the percentage of spermatozoa with intact acrosome was significantly higher than CMTG (Figures 6A–C).

### Lipid Peroxidation Analysis (TBARS Test)

**Figure 7:**
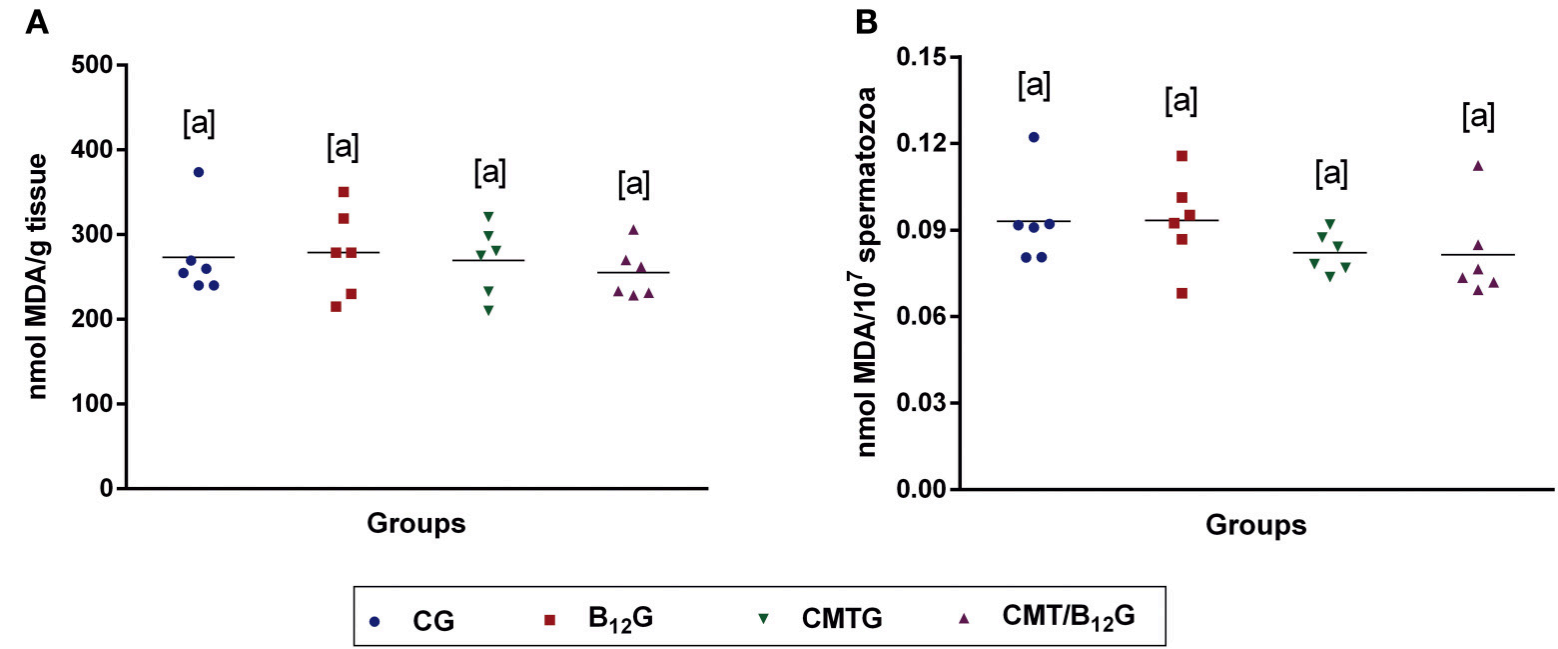
Malondialdehyde (MDA) levels in testis **(A)** and epididymal sperm **(B)** of rats from CG, B_12_G, CMTG and CMT/B_12_G. (Values expressed as mean ± SD. One-way ANOVA. *n* = 6).

No significant difference was detected in the levels of MDA either in the testicular homogenate or in the epididymal sperm among the groups (Figures 7A,B).

### Comet Assay

As shown in Table 1, the parameters evaluated by Comet assay (percentage of fragmented DNA in tail, tail length, tail moment and Olive moment) were significantly elevated in the spermatozoa of the animals from CMTG when compared with the control and B_12_ groups. However, the supplementation of the treated animals with vitamin B_12_ decreased significantly the percentage of fragmented DNA in tail when compared with CMTG. No statistical difference was detected among CG, B_12_G and CMT/B_12_G.

**Table 1:** Analysis of sperm DNA fragmentation assessed by comet assay in rats from CG, B_12_G, CMTG and CMT/B_12_G (*n* = 6).

	**Tail DNA (%)**	**Tail length (μm)**	**Tail moment**	**Olive moment**
CG	7.3 ± 3.1^a^	4.7 ± 2.0^a^	0.39 ± 0.37^a^	0.62 ± 0.34^a^
B_12_G	7.4 ± 2.2^a^	4.0 ± 0.8^a^	0.32 ± 0.14^a^	0.58 ± 0.21^a^
CMTG	15.6 ± 3.1^b^	7.3 ± 1.2^b^	1.16 ± 0.34^b^	1.26 ± 0.24^b^
CMT/B_12_G	9.3 ± 0.5^a^	5.9 ± 1.2^a, b^	0.57 ± 0.12^a^	0.84 ± 0.08^a^

*Values expressed as mean ± SD (One-way ANOVA followed by Tukey's test)*.

*The different superscript letters mean differences among the groups: a ≠ b; (p < 0.05)*.

### Fertility Evaluation

The treatment with cimetidine did not alter the parameters of reproductive outcome (number of corpora lutea, implantation, resorptions, fetuses, and weights of placenta and fetuses) in comparison with the other groups. The fertility potential was also similar among the groups (Table 2).

**Table 2:** Fertility parameters of the females (*n* = 12) naturally mated with male rats (*n* = 6) from CG, B_12_G, CMTG and CMT/B_12_G.

**Parameters**	**CG (*n* = 12)**	**B_**12**_G (*n* = 12)**	**CMTG (*n* = 12)**	**CMT/B_**12**_G (*n* = 12)**
Fertility potential (%)^a^	96.65 (91.85–100.0)	96.45 (91.7–100.0)	87.5 (79.28–92.9)	93.8 (85.13–100.0)
Pre-implantation loss (%)^a^	3.35 (0.0–8.15)	3.55 (0.0–8.3)	12.5 (7.1–20.75)	6.3 (0.0–14.88)
Post-implantation loss (%)^a^	0.0 (0.0–0.0)	0.0 (0.0–8.5)	7.4 (0.0–13.0)	2.95 (0.0–7.1)
Corpora lutea number^b^	13.8 ± 1.4	13.4 ± 1.6	13.9 ± 1.7	14.8 ± 1.7
Implantation number^b^	13.2 ± 1.2	12.8 ± 1.7	12.0 ± 2.0	13.3 ± 2.8
Resorption number^a^	0.0 (0.0–0.0)	0.0 (0.0–1.0)	1.0 (0.0–1.75)	0.5 (0.0–1.0)
Number of live fetuses^b^	12.9 ± 1.0	12.3 ± 1.6	11.2 ± 1.6	12.2 ± 2.9
Fetus weight (g)^b^	2.29 ± 0.16	2.53 ± 0.41	2.63 ± 0.41	2.44 ± 0.19
Placental weight (g)^b^	0.50 ± 0.07	0.49 ± 0.09	0.56 ± 0.05	0.54 ± 0.06

a *Values expressed as median and interquartile intervals (Kruskal-Wallis test)*.

b *Values expressed as mean ± SD (One-way ANOVA)*.

## Discussion

**Figure 8:**
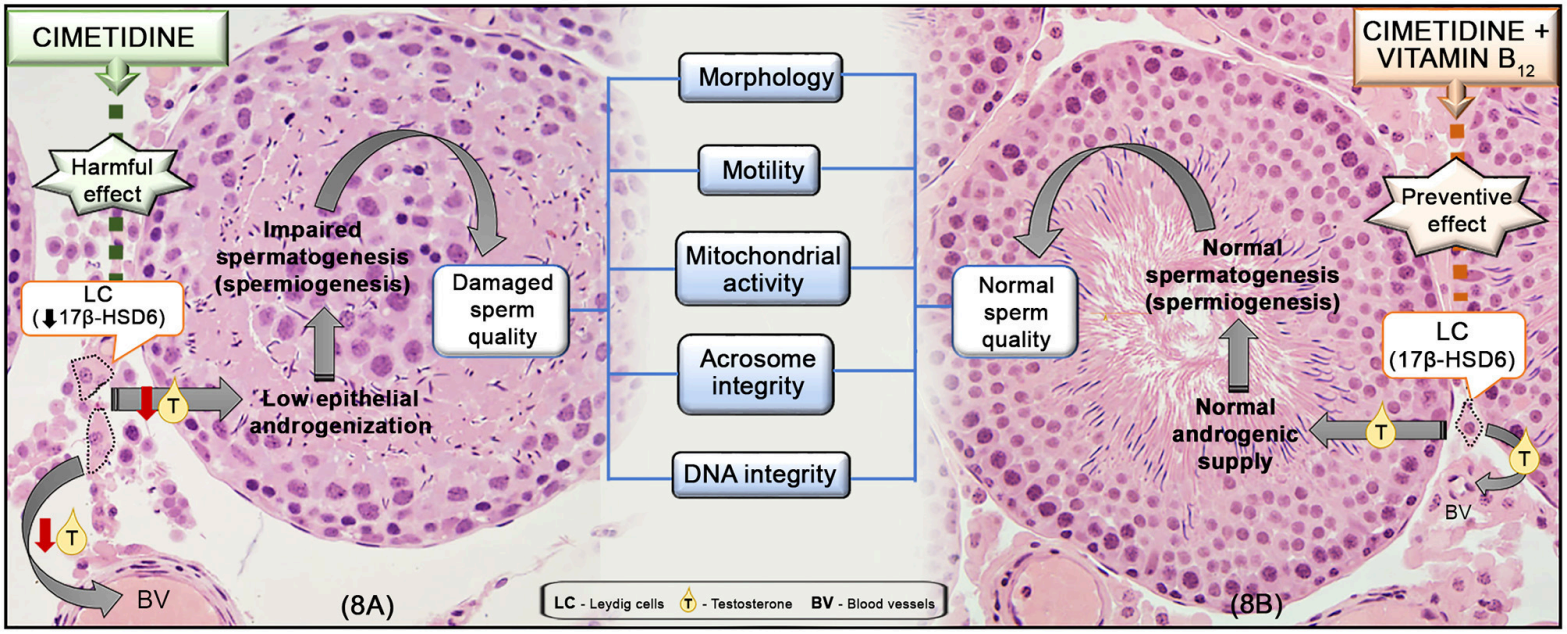
Representative scheme of the mechanism of action of cimetidine **(A)** and the preventive effect of vitamin B_12_ supplementation during the treatment **(B)** in the testes and sperm quality. In **(A)**, cimetidine impairs LC steroidogenic activity, reducing 17β-HSD6 and the serum T levels. The low androgenic supply to the seminiferous epithelium impairs spermiogenesis and, subsequently, sperm quality, including: morphology, motility, mitochondrial activity as well as acrosome and DNA integrity. In **(B)**, the supplementation of cimetidine-treated animals with vitamin B_12_ is able to prevent the harmful effect of cimetidine on the LC, maintaining the normal T levels, tubular androgenization and spermatogenic process (spermiogenesis), avoiding damage to sperm parameters.

In previous studies, we have demonstrated that cimetidine impairs the testicular structure and steroidogenesis (7, 9–11, 28, 44), causing low testosterone bioavailability and epididymal androgenic dysfunction (8). Therefore, we evaluated the impact of this androgen disrupter on sperm quality and male fertility, and whether vitamin B_12_ supplementation is able to prevent the sperm parameters changes induced by cimetidine. We also tested the hypothesis that the spermatogenic improvement induced by this vitamin can be associated with its effect on steroidogenesis. The results showed that cimetidine causes significant changes in the sperm quality. However, testosterone levels and steroidogenic activity were unchanged in the vitamin B_12_ supplemented rats, while the sperm quality was improved by the supplementation (Figure 8).

A significant increase in the flagellum abnormalities, especially tail looping or bending, was observed in spermatozoa of CMTG. These changes have been reported in spermatozoa with low or absent motility (45), and indicate failure in the spermiogenic process. Moreover, alterations in sperm motility may be related to a reduction in the expression of exclusive genes of spermatids, involved in the development of flagellar structures, such as the mitochondrial sheath of mid-piece, and required for the glycolytic enzymes function (46). Thus, the reduction of motile spermatozoa caused by cimetidine is related to the structural flagellar changes and low mitochondrial activity.

Decrease in sperm motility has also been reported in rats treated with the antiandrogenic flutamide (47). Cimetidine exerts antiandrogenic action, competing with testosterone for the androgen receptor (AR) in prostate and kidney cells (48, 49). In the epididymis, this drug reduces the stromal SHBG levels and impairs the epithelial AR nuclear translocation (8). In the present study, serum testosterone levels reduced significantly in CMTG, confirming previous findings (7, 8). Since testosterone is essential for the completion of spermiogenesis (50), the deficient androgenization induced by cimetidine may be the main cause of the flagellar abnormalities and reduction of motile spermatozoa (Figure 8). This hypothesis is reinforced by the presence of acrosomal changes in CMTG, since the acrosome formation is also an androgen-dependent process (50). It is important to emphasize that the rats received cimetidine for 52 days, but the period of spermatogenesis and the transit time taken from the testis to the cauda epididymis lasts around 65 days; thus, the spermatozoa evaluated in this study (from epididymis) were exposed to the treatment from the 14th day of spermatogenesis (at pre-leptotene primary spermatocyte stage) (revised by Hermo et al. (51)). Thus, these cells were exposed to cimetidine along meiosis I and II (for 23 days) and throughout spermiogenesis (for 15 more days), carrying and accumulating the cimetidine-induced changes for 38 days, plus the period of transit from the testis to cauda epididymis (14 days), totaling 52 days (period of treatment). Therefore, it is not surprising to find abnormal spermatozoa in the epididymis in view of the long period by which these germ cells were exposed to this androgen disrupter.

Although low levels of ROS (reactive oxygen species) are required for normal sperm function, capacitation, and acrosome reaction, elevated levels of ROS may induce DNA fragmentation and apoptosis in the germ cells and spermatozoa (52). In the current study, no difference was observed in the levels of MDA either in the testis or in the epididymal sperm, indicating that the cimetidine-induced changes in the spermatozoa are not due to increased LPO. Besides ROS, several other factors may induce DNA fragmentation, such as: deficiency of chromatin recombination and packaging during spermatogenesis, aborted apoptosis of germ cells, variations in the expression pattern of spermatic protamine and incomplete chromatin remodeling during spermiogenesis (53). Since androgenic dysfunction leads to chromatin packaging errors during spermiogenesis and loss of the protamine levels in the testis and spermatozoa (54), the increased levels of DNA damage induced by cimetidine may be due to deficient testicular androgenization.

Although the cimetidine-treated animals showed 15% of sperm DNA damage, studies have demonstrated that the cut-off point that indicate low fertility in men by alkaline Comet assay is ≥45% of sperm DNA fragmentation (55). Therefore, decreased semen quality is not necessarily associated with impaired fertility (1). In the present study, no significant reduction in embryo implantation rate or embryo loss was noted, confirming that cimetidine treatment was not able to impair the fertility potential. According to Zenick and Clegg, (56), the production of spermatozoa in rodents far exceeds the necessary quantity for fertility, and spermatogenesis must be decreased by as much as 90% in rodents so that the progeny is affected. Moreover, rats submitted to androgen deprivation after complete destruction and removal of Leydig cells by using ethane dimethane sulphonate (EDS) have maintained normal sperm output and fertility for 8–10 weeks (57). In a previous study, we verified a significant reduction (45%) in the sperm concentration of cimetidine-treated rats; thus, the remaining concentration in these animals was about 60 million of spermatozoa/mL (12), whose 75% are motile and 60% are morphologically normal, according to the present study, indicating that these cells were able to fertilize the oocytes. Even if the fertilizing spermatozoon carries some DNA damage, it is known that the oocyte is able to repair, to some extent, the damage after fertilization (58).

In spite of fertility potential was not affected by the treatment, sperm quality was significantly impaired by cimetidine treatment. Therefore, we cannot exclude a possible impact of the treatment, via paternal transgenerational inheritance, on the long term offspring health. As revised by Spadafora (59), the nucleus of epididymal spermatozoa may internalize RNA-containing nanovesicles released from the paternal somatic tissues. The RNAs are amplified and the cDNA copies are transmitted to embryos during fertilization. Thus, not only haploid genomes are delivered from germ cells during fertilization, but also the epigenomes, which can be susceptible to numerous factors, including nutritional and harmful substances. Spermatozoa carrying drug-induced DNA damage may cause aberrant epigenetic programing in early embryos and contribute to instabilities later in development (35, 60). Considering that cimetidine-treated rats presented a significant decrease in DNA integrity assessed by the Comet assay, it is recommended that further investigations are carried out on the epigenetic stability and the future consequences to the progeny fathered following cimetidine exposure.

Vitamin B_12_ exerts a potential role in spermatogenesis (12, 21–23), and sperm parameters in humans (61) and animals (12, 20, 21, 62), and has been a therapeutic agent used for the treatment of male infertility, especially oligozoospermia or asthenozoospermia (63). In previous studies, we have demonstrated beneficial effects of vitamin B_12_ on the sperm concentration and spermatogenesis of cimetidine-treated rats (11, 12). A similar effect has also been demonstrated in the seminiferous epithelium of animals receiving co-administration of the antineoplastic busulphan and vitamin B_12_ (22, 23). However, a preventive effect of vitamin on the sperm exposed to these drugs has not yet been reported.

In this study, the percentage of motile spermatozoa, the mitochondrial activity, the percentage of sperm with normal morphology, intact acrosomes, and normal DNA integrity was higher in the animals from CMT/B_12_G than in CMTG. The improvement in the acrosome, sperm tail morphology and motility indicates a beneficial effect of this vitamin on spermiogenesis (Figure 8). A protective effect of vitamin B_12_ on the integrity of the sperm membrane, acrosome (62) and motility (64, 65) has also been demonstrated in other studies.

In CMT/B_12_G, testosterone levels were similar to CG, and this result was consonant with the strong 17β-HSD6 immunofluorescence in the Leydig cells and the high levels of this enzyme in the testicular extracts in the supplemented animals, confirming the preventive effect of vitamin B_12_ against the antiandrogenic effect of cimetidine. Adult Leydig cells express high levels of a transmembrane protein *amnionless* (66, 67). This protein participates in the vitamin B_12_ transport and absorption in testes, suggesting that the steroidogenic activity seems to depend on the transport of vitamin B_12_ in Leydig cells (66). Except for a study in which rats fed with diet containing high levels of vitamin B_12_ showed high serum testosterone levels (68), studies showing preventive effect of vitamin B_12_ against androgenic disruption are inexistent in the literature. Our results showed that vitamin B_12_ is able to maintain the steroidogenic activity as well as the serum testosterone at normal levels in rats following treatment with an androgen disrupter (Figure 8). Further studies are necessary to clarify the mechanism by which this vitamin maintains steroidogenesis.

## Conclusion

Although the fertility potential was not impaired by cimetidine in rodents, this drug induced significant sperm quality changes, including DNA fragmentation. Thus, considering a possible paternal epigenetic and transgenerational inheritance, further studies focusing on the impact of cimetidine on the long term offspring health are necessary. The concomitant beneficial effect of vitamin B_12_ either in the androgenic maintenance or in the sperm parameters confirms that the cimetidine-induced androgenic failure is the main cause of spermatic changes, and points to a potential preventive role of vitamin B_12_ on the Leydig cell steroidogenesis. Therefore, vitamin B_12_ may be a potential therapeutic agent for the maintenance of sperm quality and the androgenic status of patients exposed to androgen disrupters.

## Ethics Statement

The protocol regarding the treatment of the animals used in this study was approved by the Ethical Committee for Animal Research of São Paulo Federal University-UNIFESP/EPM, Brazil (CEUA n° 7950060514) and by Ethics Committee of Dental School—São Paulo State University (UNESP), Araraquara/SP, Brazil (CEUA n° 28/2014).

## Author Contributions

ES-C coordinated the study. SM and VV contributed to the methodological design of the experiment. FB carried out the treatment of animals. FB, FdS, and ES-C collected and carried out the histological processing. RC cooperated in the processing of sperm samples. FB carried out the sperm analysis (motility and morphology, acrosome integrity, and comet assay), TBA test, fertility evaluation and immunofluorescence reaction. VV cooperated in comet assay. FdS, ES-C, and VV cooperated in fertility evaluation. FdS carried out the mitochondrial activity analysis and western blot. FB and PC carried out the immunohistochemistry reaction. VV, SM, and PC contributed in the critical analysis of results, statistical analysis and in the revision of the manuscript. FB and ES-C examined and selected the images and participated in the manuscript design. All the authors read and approved the final version of the manuscript.

### Conflict of Interest Statement

The authors declare that the research was conducted in the absence of any commercial or financial relationships that could be construed as a potential conflict of interest.
